# A Systematic Review on Novel *Mycobacterium tuberculosis* Antigens and Their Discriminatory Potential for the Diagnosis of Latent and Active Tuberculosis

**DOI:** 10.3389/fimmu.2018.02476

**Published:** 2018-11-09

**Authors:** Noëmi R. Meier, Marc Jacobsen, Tom H. M. Ottenhoff, Nicole Ritz

**Affiliations:** ^1^University of Basel Children's Hospital, Mycobacterial Research, Basel, Switzerland; ^2^University of Basel, Faculty of Medicine, Basel, Switzerland; ^3^Department of General Pediatrics, Neonatology, and Pediatric Cardiology, University Children's Hospital, Heinrich Heine University, Düsseldorf, Germany; ^4^Department of Infectious Diseases, Leiden University Medical Center, Leiden, Netherlands; ^5^The Royal Children's Hospital Melbourne, Infectious Disease Unit, Melbourne, VIC, Australia

**Keywords:** interferon gamma-release assay, immune-response, clinical studies, active tuberculosis, latent tuberculosis, cytokines

## Abstract

**Background:** Current immunodiagnostic tests for tuberculosis (TB) are based on the detection of an immune response toward mycobacterial antigens injected into the skin or following an *in-vitro* simulation in interferon gamma-release assays. Both tests have limited sensitivity and are unable to differentiate between tuberculosis infection (LTBI) and active tuberculosis disease (aTB). To overcome this, the use of novel *Mycobacterium tuberculosis* (*M. tuberculosis)* stage-specific antigens for the diagnosis of LTBI and aTB has gained interest in recent years. This review summarizes current evidence on novel antigens used for the immunodiagnosis of tuberculosis and discrimination of LTBI and aTB. In addition, results on measured biomarkers after stimulation with novel *M. tuberculosis* antigens were also reviewed.

**Methods:** A systematic literature review was performed in Pubmed, EMBASE and web of science searching articles from 2000 up until December 2017. Only articles reporting studies in humans using novel antigens were included.

**Results:** Of 1,533 articles screened 34 were included in the final analysis. A wide range of novel antigens expressed during different stages and types of LTBI and aTB have been assessed. *M. tuberculosis* antigens Rv0081, Rv1733c, Rv1737c, Rv2029c, Rv2031 and Rv2628, all encoded by the dormancy of survival regulon, were among the most widely studied antigens and showed the most promising results. These antigens have been shown to have best potential for differentiating LTBI from aTB. In addition, several studies have shown that the inclusion of cytokines other than IFN-γ can improve sensitivity.

**Conclusion:** There is limited evidence that the inclusion of novel antigens as well as the measurement of other biomarkers than IFN-γ may improve sensitivity and may lead to a discrimination of LTBI from aTB.

## Introduction

Tuberculosis (TB) was globally one of the top 10 causes of death in 2016 and more than 10 million new cases are estimated to occur each year (1). Accurate and early identification of latent tuberculosis infection (LTBI) has become one of the key strategies to reduce TB incidence in recent years (1, 2). This strategy is particularly important in those at risk for rapid progression from LTBI to active tuberculosis (aTB), which includes immunocompromised individuals and children (3).

The tuberculin skin test for many decades has been the standard immunodiagnostic test to detect LTBI, measuring the local response after intradermal injection of a purified protein derivative (4, 5). As the tuberculin skin test lacks specificity due to cross-reactivity with non-tuberculosis mycobacteria and the widely used vaccine strain *Mycobacterium bovis* Bacillus Calmette–Guérin (BCG), an *in vitro* antigen specific cytokine-based immuno-diagnostic test was developed in the 1990s (6, 7). The currently used interferon gamma-release assays include early secretory antigenic target (ESAT)-6 and culture filtrate protein (CFP)-10 as stimulatory antigens. Both antigens are located in the region of difference 1, which is absent in BCG and in most non-tuberculosis mycobacteria rendering these tests more specific than the tuberculin skin test (8, 9). Interferon gamma-release assays have now in many setting replaced the tuberculin skin test for testing LTBI in adults and have been recommended for use in both resource-rich and resource-limited countries (10). Interferon gamma-release assays have two major limitations: limited sensitivity in children, particularly in those under 5 years of age, and inability to discriminate between LTBI and aTB (11–13).

Numerous studies have therefore aimed to identify other proteins from *Mycobacterium tuberculosis* (*M. tuberculosis*) different from ESAT-6 and CFP-10 as potential candidates for inclusion in immunodiagnostic tests for TB. Several strategies employing *in vitro, in vivo*, and *in silico* approaches have led to the discovery of novel immunogenic proteins of *M. tuberculosis* (14–16). An important milestone and the basis for these discoveries was the sequencing of the entire genome of *M. tuberculosis* in the late 90ies (17). To summarize the current state of research, we performed a systematic literature review on studies in humans that have included novel *M. tuberculosis* antigens for immunodiagnostic tests. The aim of this review was to compare current evidence on novel *M. tuberculosis* antigens for the diagnosis of LTBI and aTB and to identify antigens that are most promising to be included in further research. In addition, results on measured biomarkers after stimulation with novel *M. tuberculosis* antigens were also reviewed.

## Methods

### Search strategy

A systematic literature search was performed guided by preferred reporting items for systematic reviews and meta-analyses (see PRISMA checklist Supplementary File 1). Studies measuring the immune response induced by novel *M. tuberculosis* antigens in patients with LTBI and aTB were included. PubMed, Embase and Web of Science (core collection) were searched for studies published between Jan 1st 2000 and Dec 31st 2017. Studies published before 2000 were excluded since interferon gamma-release assays have been marketed in the late 1990s. The PubMed search was done using medical subject heading terms. Search strings were generated for (i) tuberculosis, (ii) antigens, (iii) assay read-out and (iv) humans. Additional filters were included in the Embase and Web of science search (see Supplementary File 2 for details on search strategy). Articles in English, French or German were considered for inclusion. Additional studies were identified through references of publications identified for inclusion.

### Inclusion and exclusion criteria

**Figure 1:**
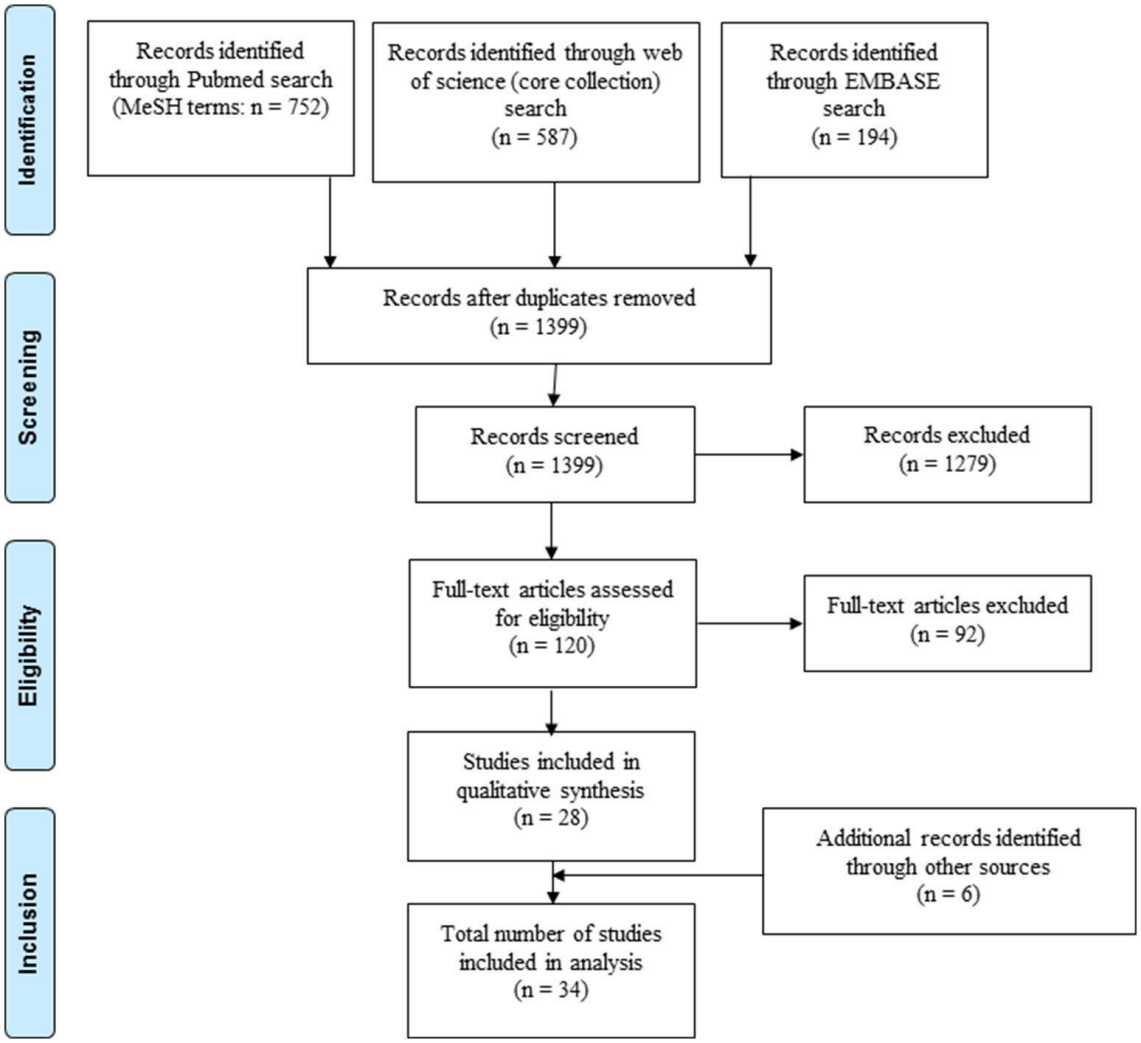
Selection of articles included in the review.

After initial screening (Figure 1) studies were evaluated for inclusion based on the following criteria (see also Supplementary File 3): (i) including patients with LTBI and/or aTB with details specified on classification of included patients comprising results from sputum microscopy, culture, polymerase chain reaction testing, radiography, and standard immunodiagnostic tests (interferon gamma-release assays or tuberculin skin test); (ii) further details of the study population available including age, gender, treatment, and testing for human immunodeficiency virus, (iii) immune response analyzed in blood samples for at least one novel antigen not currently or previously used in commercially available interferon gamma-release assays or tuberculin skin test (i.e. antigen other than ESAT-6, CFP-10, Tb7.7, purified protein derivative). If human immunodeficiency virus positive individuals were included, separate analysis of results from this patient group was required for inclusion. For pediatric studies, authors were contacted, if human immunodeficiency virus status was not specified in the original publication. Pediatric studies were included if children were human immunodeficiency virus negative or if human immunodeficiency virus was highly unlikely based on national prevalence of the study population. Studies including patients that have been treated with anti-tuberculous medication for 4 weeks or longer prior to testing were excluded from analysis. The search and selection of included studies was done by two authors (NM and NR). In unclear cases a shared decision for inclusion of the study was made.

### Data extraction and classification

Data was extracted using a standardized form including the following variables: First author, year of publication; country; study population characteristics including age, patient group; details about methods used including cell type, incubation duration, type of antigen, assays used for read-out and main results. According to function, expression and location on the genome antigens were grouped into latency associated, reactivation- and resuscitation- associated or other.

## Results

### Included studies

A total of 1,533 records were identified through PubMed, Embase and Web of Science, of which 28 studies were included in the analysis (Figure 1). An additional six records were identified through cross-references of included articles resulting in a final number of 34 included studies. Reasons for exclusion of studies are summarized in Supplementary File 4.

### Characteristics of included studies

Included studies originated from 16 countries with the majority (12/34, 35%) being from Europe (Table 1). A total of 5,084 patients were included of which 2,325 were classified as LTBI and 1,252 as aTB. In addition 1,507 healthy controls or other controls were included. The majority of studies were in adults only (24/34, 70%;) (18, 20, 21, 23–27, 29–32, 35, 36, 38, 39, 42, 45–51), 8/34 (24%) studies were done in adults and children (19, 22, 28, 37, 40, 41, 43, 44) and 2/34 (6 %) were done in children only (33, 34). Outcomes were measured using enzyme-linked immunosorbent assay (ELISA), enzyme-linked immuno-spot assay (ELISPOT) and flow cytometry (FCM) in 27, 6 and 12 studies, respectively (Table 1), and some studies used several different assays for outcome measurement. IFN-γ was measured as the only outcome in 16/34 (47%) studies. 18/34 (53%) studies included further cytokines in the analysis most commonly being IL-2, IL-10, IL-17, and TNF-α (Table 2).

**Table 1:** Summary of studies included in the review.

**References**	**Year**	**Country**	**Study population**					**Assay**			
			**Total n**	**Age group**	**n aTB (form)**	**n LTBI / exp**	**n control**	**Cell type**	**Incubation time**	**Type of antigen**	**Technique**
Alvarez-Corrales et al. (18)	2013	Honduras	148	A	38 (PTB)	29 (EXP HCW)	81 (NTBC)	WB	7 d	pro, pep	ELISA
Antas et al. (19)	2005	Brazil	61	A	34 (PTB)	–	10 (HC)	PBMC	5 d	pro	ELISA
				C	9 (pTB)		8 (NTBC)		24 h, 72 h	pro	FCM
Araujo et al. (20)	2015	Brazil	71	A	7 (PTB)	47 (LTBI)	–	PBMC	5 d	pro	ELISA
						17 (EXP)					
Arroyo et al. (21)	2016	Colombia	42	A	20 (PTB)	22 (lt LTBI)	–	PBMC	7 d	pro	FCM
Bai et al. (22)	2016	China	376	A	116 (ns)	51 (LTBI)	103 (HC)	PBMC	18-20 h	pro	ELISPOT
				C			57 (NTBC)				
							49 (BCG HC)				
Belay et al. (23)	2015	Ethiopia	363	A	147 (PTB)	148 (HHC)	68 (HC)	WB	2 d	ns	ELISA
Bertholet et al. (24)	2011	USA	13	A	6 (PTB)	–	7 (HC)	WB	20-22 h	pro	ELISA
Chiacchio et al. (25)	2017	Italy	49	A	13 (ns)	9 (LTBI)	–	WB	16-20 h	pro	ELISA
					12 (ns, human immunodeficiency virus^+^)	15 (LTBI-human immunodeficiency virus^+^)		PBMC	16 h	pro	FCM
Chegou et al. (26)	2012	South Africa	124	A	23 (PTB)	101 (HHC)	–	WB	7 d	pro, pep	ELISA
Chegou et al. (27)	2012	South Africa	43	A	15 (PTB)	28 (HHC)	–	WB	7 d	pro	ELISA
Chen et al. (28)	2009	China	111	A	58 (PTB)	21 (LTBI)	32 (HC)	PBMC	22-24 h	pro	ELISPOT
				C							
Commandeur et al. (29)	2011	Norway	24	A	–	13 (lt LTBI)	11 (HC)	PBMC	6 d	pro, pep	ELISA
									16 h	pro, pep	FCM
Delogu et al. (30)	2011	Italy	87	A	26 (PTB)	19 (recLTBI)	–	WB	20-24 h, 7 d	pro	ELISA
					18 (past PTB)	24 (rLTBI)			o/n	pro	FCM
Delogu et al. (31)	2016	Italy	41	A	18 (PTB-human immunodeficiency virus^+^)	23 (LTBI-human immunodeficiency virus^+^)	–	WB	20-24 h	pro	ELISA
Doddam et al. (32)	2017	India	129	A	40 (PTB)	52 (LTBI)	37 (HC)	PBMC	24 h	pro	ELISA
Dosanjh et al. (33)	2011	Turkey	846	C	–	846 (HHC)	–	PBMC	o/n	pep	ELISPOT
Dreesman et al. (34)	2017	Belgium	61	C	15 (ns)	19 (LTBI)	27 (HC)	PBMC	5 d	pro	FCM
Goletti et al. (35)	2010	Italy	149	A	50 (PTB,EPTB)	23 (recLTBI)	15 (HC)	WB	24 h, 7 d	pro	ELISA
					45 (past PTB)	16 (rLTBI)					
Govender et al. (36)	2010	South Africa	21	A	–	21 (LTBI)	–	WB, PBMC	6 d	pep	ELISA
			50		25 (PTB)	25 (LTBI)	–	PBMC	6 d	pep	FCM
Hougardy et al. (37)	2007	Belgium	203	A	58 (PTB)	32 (recLTBI)	51 (HC)	PBMC	4 d	pro	ELISA
				C	31 (EPTB)	31 (rLTBI)					
Hozumi et al. (38)	2013	Japan	37	A	12 (PTB)	14 (LTBI)	11 (HC)	PBMC	18 h	pro	ELISPOT
Kassa et al. (39)	2012	Ethiopia	34	A	34 (PTB)	–	–	WB	7 d	pro	ELISA
Li et al. (40)	2017	China	300	A	118 (PTB)	–	55 (HC)	PBMC	18 - 20 h	pep	ELISPOT
				C	37 (EPTB)		90 (NTBC)				
Loxton et al. (41)	2012	South Africa	25	A	5 (PTB)	16 (HHC)	–	WB	7 d	pro	ELISA
				C		4 (human immunodeficiency virus^+^)					
			22		11 (PTB)	11 (HHC)	–	PBMC	16 h	pro	FCM
Mensah et al. (42)	2014	Ghana	20	A	20 (PTB)	–	–	PBMC	6 d	pro	ELISA, FCM
Michelsen et al. (43)	2016	Greenland	978	A	–	220 (recLTBI)	691 (HC)	WB	7 d	pep	ELISA
				C		67 (rLTBI)					
Michelsen et al. (44)	2017	Greenland	65	A	–	22 (recLTBI)	–	WB	7 d		ELISA
				C		32 (rLTBI) 11 (undet INF)					
Pathakumari et al. (45)	2015	India	74	A	39 (PTB)	35 (LTBI)	–	WB	6 d	pro, pep	ELISA
Pathakumari et al. (46)	2015	India	74	A	39 (PTB)	35 (LTBI)	–	WB	6 d	pro	ELISA
Peña et al. (47)	2015	Argentina	172	A	56 (PTB)	56 (LTBI)	60 (HC)	WB, PBMC	24 h, 5 d	pro, pep	ELISA, FCM
								PBMC	4d	pro, pep	FCM
Satchidanandam et al. (48)	2016	India	53	A	20 (PTB)	28 (LTBI)	5 (HC)	PBMC	48 h, 72 h	pro, pep	ELISA
								WB	18 h	pro, pep	FCM
Schwander et al. (49)	2000	USA (Mexico)	25	A	–	10 (LTBI)	15 (HC)	PBMC	24 - 72 h	pro	ELISA ELISPOT
Wyndham-Thomas et al. (50)	2014	Belgium	131	A	23 (ns)	9 (recLTBI)	24 (HC)	PBMC	24 h, 96 h	pro	ELISA, FCM
						14 (rLTBI)					
						9 (LTBI)					
						52 (undet INF)					
Wyndham-Thomas et al. (51)	2015	Belgium	62	A	14 (human immunodeficiency virus^+^)	48 (human immunodeficiency virus^+^, EXP)	–	PBMC	24 h	pro	ELISA
Total			5084		1252	2325	1507

*A, Adult; C, child*.

*PTB, pulmonary tuberculosis; EPTB, extrapulmonary TB; pTB, pleural TB*.

*ltLTBI, long-term LTBI; recLTBI, recent latent tuberculosis infection; rLTBI, remote latent tuberculosis infection*.

*undet INF, undeterminate M.tuberculosis infection; uk, unknown; HC, healthy control; HHC, house hold contact*.

*WB, whole blood; PBMC, peripheral blood mononuclear cell*.

*o/n, overnight; d, days; h, hours*.

*pro, protein; pep, peptide*.

*ELISA, enzyme-linked immunosorbent assay; ELISPOT, enzyme-linked immuno-spot assay; FCM, flow cytometry*.

**Table 2:**
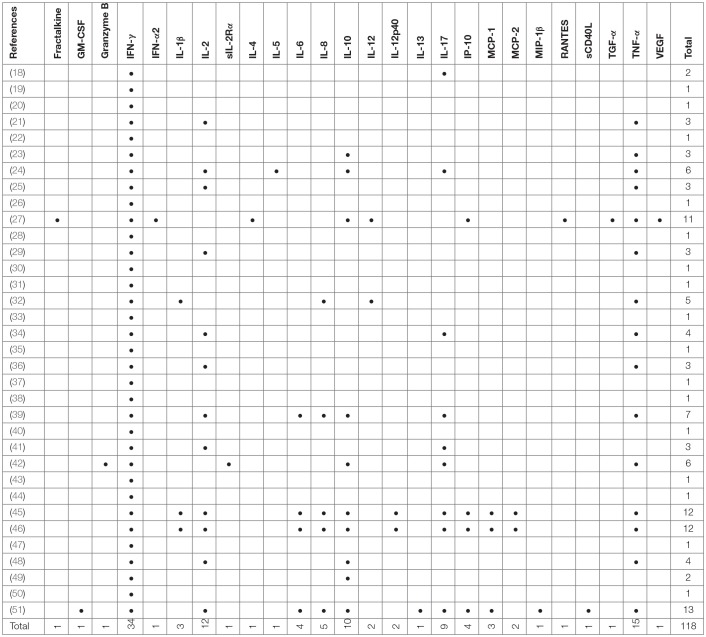
Summary of cytokines measured in studies included in this review (all cytokines listed).

### Patient groups investigated

Patient groups in the studies included aTB, LTBI, exposed, healthy, and sick control patients (Tables 1, 3). Criteria for patients with aTB were similar and mostly consistent across studies including culture confirmation and/or presence of acid fast bacilli in sputum smear. Two studies also used positive polymerase chain reaction results as confirmation of aTB (34, 37). Further to this in 6/34 (18%) studies diagnosis of aTB was based on clinical criteria (20, 34, 37, 40, 50, 51). Definitions for LTBI patients were heterogeneous and highly variable across studies. Overall 14/34 (41%) studies included a control group of healthy participants who had negative test results for interferon gamma-release assays /tuberculin skin test and/or no known history of or exposure to aTB (19, 22, 24, 28, 29, 32, 34, 35, 37, 38, 43, 47, 48, 50). A further 2/34 (6%) studies included exposed individuals into the control group as long as they stayed healthy (e.g., no symptoms and microbiologically negative or tuberculin skin test negative) (32, 34). Another 2/34 (6%) studies included a healthy control group but either no interferon gamma-release assays /tuberculin skin test was done or the community controls were tuberculin skin test positive (no exposure) (40, 49). Finally, 1/34 (3%) study included patients with a respiratory disease other than TB as a control group (18) and 3/34 (9%) included both a healthy and a sick control group (19, 22, 40). A single patient group was evaluated by 4/34 (12%) studies being either: active pulmonary TB (39, 42), pediatric household contacts from TB index cases (33), or LTBI patients (44).

**Table 3:** Definitions of patient groups included in the studies.

**References**	**TB group**	**Exposed and/or LTBI**			**Controls (healthy)**			**Controls (other disease)**
		**Exposure type**	**Interferon gamma-release assays /tuberculin skin test results**	**Other**	**Exposure**	**Interferon gamma-release assays /tuberculin skin test results**	**Other**
(18)	Smear or culture positive	Exposed healthcare workers	ns	Asymptomatic, culture negative, AFS negative	–	–	–	Respiratory symptomatic patients but not TB, smear and culture negative
(19)	Smear positive	–	–	–	–	Tuberculin skin test negative	No known history of TB	Non-TB pulmonary diseases
(20)	Smear and/or culture positive and/or Clinical suspicion	Exposed (recent close contacts)	Interferon gamma-release assays and tuberculin skin test status (interferon gamma-release assays +, tuberculin skin test +, DP, DN)	–	–	–		–
(21)	Culture positive	Household contacts of index case (in the past)	Positive IFN-γ response towards CFP-10	Asymptomatic for long period [5-7 years], living in endemic area	–	–	–	–
(22)	Clinical suspicion and suggestive X-ray optional Smear or culture positive, strongly tuberculin skin test positive	–	rESAT-6/CFP-10 fusion protein positive (ELISPOT assay)	Asymptomatic	–	rESAT-6/CFP-10 fusion protein (ELISPOT assay) negative	Healthy, BCG vaccinated	Non-TB respiratory disease (TB excluded)
(23)	Smear positive (min 2)	Household contacts of index case	ns	Smear negative (if productive cough)	No exposure	–	No known history of TB	–
(24)	Culture positive and Tuberculin skin test positive (>10 mm)	–	–	–	–	Tuberculin skin test negative	Not BCG vaccinated	–
(25)	Culture positive	–	QFT positive	Asymptomatic, normal X-ray, smear or culture negative	–	–	–	–
(26)	Smear positive (min 2)	Household contacts of index case	Not done	Asymptomatic, normal X-ray, smear or culture negative	–	–	–	–
(27)	Smear positive (min 2)	Household contacts of index case	Not done	Normal X-ray, smear negative (min 2)	–	–	–	–
(28)	Clinical suspicion and Smear or culture positive or suggestive X-ray	Household contacts of index case	T-SPOT.TB positive	Asymptomatic, normal X-ray	No exposure	T-SPOT.TB negative	No known history of TB, BCG vaccinated	–
(29)	–	Exposed to index case (in the past)	Tuberculin skin test positive (>10 mm)	–	–	Purified protein derivative negative	Healthy	–
(30)	aTB: culture positive or M.tb Gen-probe positive and QFT positive past TB: culture positive (documented), successfully treated, QFT positive	Close contacts / household contacts of index case (remote: within the last 3 years; recent: within the last 3 months)	QFT and tuberculin skin test positive (>5 mm close contact; ≥10 mm others)	–	–	–	–	–
(31)	Smear and culture positive	–	QFT-GIT positive	Asymptomatic, normal X-ray	–	–	–	–
(32)	Smear and culture positive and Suggestive X-ray	Exposed (healthcare workers or exposure to index case)	QFT positive	Asymptomatic, normal X-ray, smear negative	Exposed (healthcare workers or exposure to index case)	QFT negative	Healthy, normal X-ray, smear negative	–
(33)	Index case: smear positive	Household contacts of index case	ns	–	–	–	–	–
(34)	Exposed to index case and Clinical suspicion and Suggestive X-ray and Tuberculin skin test positive (≥5 mm), Optional culture or polymerase chain reaction positive	Exposure to index case	Tuberculin skin test positive (≥5 mm; ≥15 mm for BCG vaccinated)	Asymptomatic, normal X-ray	Exposure to index case	Tuberculin skin test negative (up to 8-12 weeks after last contact with index case)	Healthy	–
(35)	aTB: culture positive or M.tb Gen-probe positive Cured TB: culture positive (documented) and Successfully treated	Household contacts of index case (remote: within the last 3 years; recent: within the last 3 months)	QFT and tuberculin skin test positive (≥5 mm recent; ≥10 mm remote)	Normal X-ray (recent LTBI)	–	QFT negative and tuberculin skin test negative	No risk of M.TB infection	–
(36)	Clinical suspicion and Smear or culture positive	–	ELISPOT or QFT-GIT or tuberculin skin test (>10 mm) positive (for more than a year)	Asymptomatic	–	–	–	–
(37)	Clinical suspicion and/or Culture positive and/or Smear positive and/or Polymerase chain reaction positive and/or Presence of granuloma in biopsy	Household contacts of index case, healthcare workers and others (recent LTBI: conversion within 2 years; remote LTBI: conversion after 2 years)	tuberculin skin test positive (>10 mm)	Subgroups based on tuberculin skin test conversion	No exposure	Tuberculin skin test negative	No known history of TB	–
(38)	Smear and culture positive	–	QFT positive	Asymptomatic, normal X-ray, culture negative	No exposure	QFT negative	Healthy, no known history of TB, normal X-ray, BCG vaccinated	–
(39)	Smear positive (min 2)	–	–	–	–	–	–	–
(40)	Clinical suspicion and/or Suggestive x-ray and/or Culture positive	–	–	–	–	–	Healthy, no known history of TB, normal X-ray	Non-TB pulmonary disease (active TB excluded)
(41)	Culture positive	Household contacts of index case	ns	Asymptomatic, normal X-ray, culture negative	–	–	–	–
(42)	Smear positive	–	–	–	–	–	–	–
(43)	–	–	QFT done	Recent LTBI: QFT positive; remote LTBI: prior notified TB	–	QFT negative	No prior notified TB	–
(44)	–	–	QFT done	Recent LTBI: QFT positive; remote LTBI: prior notified TB; undetectable LTBI: QFT negative	–	–	–	–
(45)	Smear positive [3 times] and qft-git positive	Household contacts of index case (at least 3 months)	QFT-GIT positive	Asymptomatic, normal X-ray, smear negative	–	–	–	–
(46)	Smear positive [3 times] and qft-git positive	Household contacts of index case (at least 3 months)	QFT-GIT positive	Asymptomatic, normal X-ray, smear negative	–	–	–	–
(47)	Smear and culture positive and Clinical and radiological confirmation of tb	Household contacts of index case and healthcare workers	QFT-GIT positive and/or tuberculin skin test positive (>10 mm)	Asymptomatic, normal X-ray	–	QFT negative and tuberculin skin test negative	Normal X-ray, smear negative	–
(48)	Culture positive	–	Tuberculin skin test positive (>9 mm), IFN-γ response (≥ 0.7 IU) to peptide pool of ESAT-6 and CFP-10	Asymptomatic, smear negative	–	Tuberculin skin test negative (<5 mm), no IFN-γ response to ESAT-6 & CFP-10 peptides	–	–
(49)	–	Household contacts of index case (smear grade 3) for at least 3 months	Tuberculin skin test positive (>10 mm)	Asymptomatic, normal X-ray	No exposure	Tuberculin skin test positive (>10 mm)	Healthy, normal X-ray	–
(50)	Clinical suspicion and/or Culture positive	Risk of exposure defined (remote LTBI: tuberculin skin test conversion and/or TB contact ≥2 years ago; recent LTBI: tuberculin skin test conversion and/or TB contact within the last 2 years; undeterminate LTBI: no active TB but tuberculin skin test results doubtful	Tuberculin skin test positive (>10 mm)	Combination of X-ray, tuberculin skin test and exposure	–	Tuberculin skin test negative	–	–
(51)	Clinical suspicion and/or Culture positive	Risk of exposure defined	QFT and tuberculin skin test done	Combination of X-ray, QFT and tuberculin skin test results and exposure risk	–	–	–	–

*ns, not specified*.

*DP, double positive; DN, double negative*.

### Types of antigens investigated

Over 300 individual novel M. tuberculosis antigens were tested among the studies included in this review. Table 4 shows a selection of the most commonly reported 92 antigens that were reported in these studies. Of those Rv2031c, Rv2029c, antigens of the Ag85 complex, Rv0475 (HBHA), Rv2628, Rv1733c, Rv1737c, Rv0081, Rv2032, Rv0867c, and Rv2389c are among the most frequently tested ones. Most antigens tested (48 antigens; Rv2031c, Rv2029c, Rv2628, Rv1733c, Rv1737c, Rv0081, Rv2032) belong to the group of latency associated antigens. The majority of these 42/48 (88%) are part of the dormancy of survival regulon (DosR), a region in the genome comprising approximately 50 genes expressed during latency. Fewer antigens (e.g., Rv0867c, Rv2389c) belong to the group of resuscitation associated antigens, which contains 5 different genes encoding resuscitation promoting factors (Rpfs). Table 5 summarizes the most signicant findings for the most important antigens assessed in the studies grouped according to family of antigens.

**Table 4:**
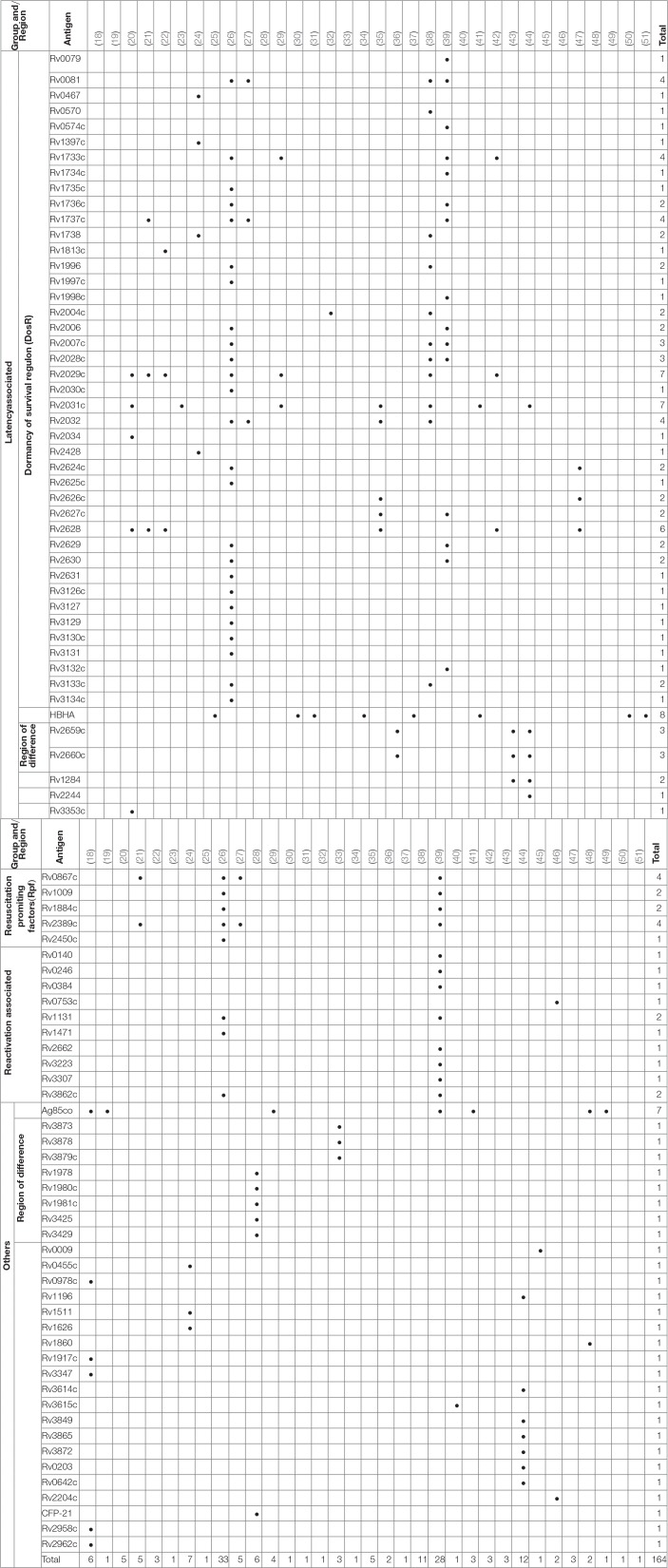
Summary of antigens used in the studies included in this review.

**Table 5:** Summary of significant findings of most important antigens assessed in studies grouped according to antigen families.

**References**	**Latency associated antigens**							**Resuscitation promoting factors (Rpfs)**		**Others**
	**Dormancy of survival regulon (DosR)**								
	**Rv0081**	**Rv1733c**	**Rv1737c**	**Rv2029c**	**Rv2031c**	**Rv2628**	**HBHA**	**Rv0867c**	**Rv2389c**	**Ag85complex**
(18)	NA	NA	NA	NA	NA	NA	NA	NA	NA	Higher concentrations of IFN-γ and Il-17 in exp vs. aTB patients
(19)	NA	NA	NA	NA	NA	NA	NA	NA	NA	Higher concentration of IFN-γ in treated aTB patients & community controls vs. untreated patients
(20)	NA	NA	NA	Higher concentrations of IFN-γ in LTBI vs. aTB patients & controls			NA	NA	NA	NA
(21)	NA	NA	Higher proportion of IFN-γ and/or TNF-α-producing CD4 & CD8 T cells in LTBI vs. aTB patients		NA	NS	NA	NS	Higher proportion of IFN-γ and/or TNF-α-producing CD4 or CD8 T cells in LTBI vs. aTB patients	NA
(22)	NA	NA	NA	Higher concentrations of IFN-γ in LTBI vs. aTB patients & controls	NA	Higher concentrations of IFN-γ in LTBI vs. aTB patients & controls	NA	NA	NA	NA
(23)	NA	NA	NA	NA	Lower concentrations of IFN-γ, Il-10 & TNF-α in aTB patients vs. controls	NA	NA	NA	NA	NA
(25)	NA	NA	NA	NA	NA	NA	Lower concentrations of IFN-γ in HIV^+^ individuals with aTB & LTBI compared to HIV^−^ patients	NA	NA	NA
(26)	Higher concentrations of IFN-γ in exp vs. aTB patients		NS	NS	NA	NA	NA	Higher concentrations of IFN-γ in exp vs. aTB patients		NA
(27)	Higher concentrations of Il-10, Il-12(p40), IP-10 & TNF-α in exp vs. aTB patients	NA	Higher concentrations of Il-10, Il-12(p40), IP-10 & TNF-α in exp vs. aTB patients	NA	NA	NA	NA	NS	Higher concentrations of TGF-α in aTB patients vs. exp	NA
(29)	NA	Higher proportion of IFN-γ/TNF-α-producing CD8 T cells in LTBI vs. healthy controls	NA	Higher proportion of IFN-γ/TNF-α-producing CD8 T cells in LTBI vs. healthy controls		NA	NA	NA	NA	NS
(30)	NA	NA	NA	NA	NA	NA	Higher concentration of IFN-γ in LTBI vs. aTB	NA	NA	NA
(31)	NA	NA	NA	NA	NA	NA	Low concentrations of IFN-γ in LTBI & aTB patients infected with HIV	NA	NA	NA
(34)	NA	NA	NA	NA	NA	NA	Higher proportion of CD4 T cells producing one or more cytokines in LTBI & aTB patients compared to healthy controls	NA	NA	NA
(35)	NA	NA	NA	NA	NS	Higher concentrations of IFN-γ in remote LTBI vs. aTB patients, recent LTBI & controls	NA	NA	NA	NA
(37)	NA	NA	NA	NA	NA	NA	Higher concentrations of IFN-γ in LTBI vs. aTB patients	NA	NA	NA
(38)	NS	NA	NA	Higher proportion of IFN-γproducing T cells in LTBI vs. aTB patients	NS	NA	NA	NA	NA	NA
(39)	High concentrations of Il-6, Il-10 & TNF-α in aTB		NS	NA	NA	NA	NA	High concentrations of TNF-α, Il-10 & Il-6 in aTB		NS
(41)	NA	NA	NA	NA	NS	NA	Higher proportion of IFN-γ/Il-2/Il-17-producing CD4 T cells in household contacts vs. aTB patients	NA	NA	NS
(42)	NA	Increase of Granzyme B, IFN-γ, Il-17 & sIl-2Rα concentrations after treatment of aTB patients	NA	Increase of IFN-γ, granzyem B, Il-17 & sIl-2Rα levels after treatment of aTB patients	NA	Increase of IFN-γ, granzyem B, Il-17 & sIl-2Rα levels after treatment of aTB patients	NA	NA	NA	NA
(47)	NA	NA	NA	NA	NA	NS	NA	NA	NA	NA
(49)	NA	NA	NA	NA	NA	NA	NA	NA	NA	Higher proportions of IFN-γ producing T cells in household contacts vs. controls
(50)	NA	NA	NA	NA	NA	NA	Higher concentration of IFN-γ in LTBI vs. aTB patients and controls	NA	NA	NA
(51)	NA	NA	NA	NA	NA	NA	Higher concentrations of IFN-γ compared to 12 other cytokines in HIV^+^ patients (with LTBI/aTB)	NA	NA	NA

### Latency associated antigens

#### Dormancy of survival regulon

Two studies by Chegou et al. and Kassa et al. assessed the immune response against a wide range of stage-specific antigens, including serval antigens of the DosR regulon (26, 39). Both studies used stimulated whole blood to assess IFN-γ response in a long-term assay. Kassa et al. identified Rv0081, Rv1733c and Rv2006 among the most immunogenic antigens stimulating high concentrations of TNF-α, IL-10, and IL-6 in aTB (39). Chegou et al. reported significant differences in IFN-γ responses for a number of DosR antigens including Rv1735c, Rv2006, Rv2625c, Rv1996, Rv2032, Rv2629, Rv3126c, Rv0081, Rv2631, Rv3130c, Rv2624c, Rv2007c, Rv2028c, and Rv3134 between exposed and aTB (26). Based on these results a selection of these antigens was tested in a further study which also measured additional cytokines. Stimulation with Rv0081, Rv2032, and Rv1737c had the highest discriminatory potential of aTB vs. exposed when IL-12(p40), IP-10, IL-10 and TNF-α were analyzed (27). A study by Mensah et al. evaluated the immune response after stimulation with Rv1733c, Rv2029c, and Rv2628 to monitor treatment response in aTB patients (42). Concentrations of several biomarkers including IFN-γ, Granzyme B, IL-17, and sIL-2Rα increased during anti-mycobacterial treatment but only Rv1733c-specific Granzyme B levels were significantly increased compared to baseline levels. Hozumi et al. found 6 out of 33 tested DosR antigens (Rv0570, Rv1996, Rv2004c, Rv2028c, Rv2029c, and Rv3133c) relevant for differentiation of aTB and LTBI as these induced higher concentrations of IFN-γ in LTBI (38).

Other DosR antigens such as Rv2031c showed conflicting results. For example, Belay et al. found that short-term stimulation with Rv2031c resulted in significantly lower IFN-γ, TNF-α, and IL-10 concentrations in aTB patients compared to TB exposed individuals and healthy controls at baseline and over a 12-month follow-up period (23). Conversely, Goletti et al. and Hozumi et al. did not find differences in IFN-γ concentrations upon Rv2031c stimulation in aTB, LTBI patients and controls (35, 38). Loxton et al. looked at IFN-γ concentrations in 7-days Rv2031c-stimulated whole blood and found no differences between aTB and household contacts (41).

Commandeur et al. showed stimulation with Rv1733c, Rv2029c and Rv2031c resulted in an increase of double and single cytokine-producing T cells among LTBI patients compared to healthy controls. Particularly IFN-γ/TNF-α-producing CD8 T cells were the most frequently found subset (29). This supports findings from a study by Arroyo et al. that reported stimulation with Rv1737c and Rv2029c increased IFN-γ- and/or TNF-α-producing CD4 and CD8 T cells in patients with LTBI compared to aTB (21). IFN-γ production in response to Rv2029c was also significantly increased in LTBI compared to aTB patients and healthy controls in a study by Bai et al. (22). This study also included a control group with respiratory disease and showed that the response to latency antigens Rv2029c, Rv2628, Rv1813c was negligible in these patients. Araujo et al. used Rv2029c, Rv2031c and Rv2034 and found an increased IFN-γ response upon stimulation in the LTBI group; the three antigens combined were able to detect 95% of LTBI patients (20).

Doddam et al. characterized the immune-response toward Rv2004c and found that this antigen elicits a strong pro-inflammatory (TNF-α, IL-8, IL-1β, and IL-12) response in LTBI patients compared to aTB and healthy controls (32). Similarly, Hozumi et al. showed that Rv2004c elicits increased IFN-γ producing T cells in a population of LTBI patients compared to aTB and controls in Japan (38).

Response to latency antigens Rv2624-30 were assessed in several studies. Goletti et al. found stimulation with Rv2628 to result in high concentrations of IFN-γ which the authors associated with protection against aTB in those patients. Rv2626c and Rv2627c, however, showed no significant differences in LTBI vs. aTB patients (35). Araujo et al. and Bai et al. also measured higher IFN-γ responses in peripheral blood mononuclear cells stimulated with Rv2628 in LTBI compared to aTB patients and healthy controls (20, 22). In contrast, results from Peña et al. suggest Rv2626c, but not Rv2624c and Rv2628 to be a strong inducers of IFN-γ in LTBI compared to aTB patients and healthy controls (47). Chegou et al. demonstrated that Rv2624c as well as Rv2625c induced significantly higher IFN-γ responses in exposed household contacts compared to aTB patients (26). In a study by Kassa et al, in aTB patients Rv2627c, Rv2629, and Rv2630 were among the most immunogenic antigens inducing high concentrations of several cytokines (including TNF-α, IL-6, IL-10) (39). Bertholet et al. assessed the effect of anti-mycobacterial treatment in aTB patients and found generally low levels of IFN-γ in response after treatment initiation. However, Rv2624 was among the few antigens that showed an increase in stimulated IFN-γ concentrations over the course of the treatment (24).

### Heparin-binding hemagglutinin (HBHA, Rv0475)

A study by Loxton et al. measuring intracellular cytokines after stimulation with HBHA found IFN-γ/IL-2/IL-17-producing CD4 T cells to be increased in household contacts compared to aTB. Interestingly, in the same study IFN-γ concentrations measured in the supernatant of a 7-day stimulation assay using HBHA did not differ among both groups (41). Similarly, Delogu et al. tested recombinant methylated HBHA in patients with LTBI and aTB and found an increased IFN-γ response in patients with LTBI in both short-term and long-term stimulation assays (30). Likewise Hougardy et al. were able to show improved sensitivity compared to ESAT-6 and purified protein derivative for identification of LTBI in a low-endemic setting using a 4-day stimulation with HBHA (37). Wyndham-Thomas et al. explored the potential of a 24 h and 96 h stimulation with HBHA for LTBI diagnosis in a low-TB-endemic setting and confirmed the diagnostic potential of the shorter incubation in detecting recent and remote LTBI compared to a commercial interferon gamma-release assays (50). In another study by the same authors including human immunodeficiency virus-infected patients they found a 24 h stimulation with HBHA induced higher concentrations of IFN-γ compared to 12 other cytokines measured (51). A further study including human immunodeficiency virus-infected patients by Delogu et al. showed that the absence of response toward HBHA correlated with increased risk of developing aTB indicating that a response to HBHA may be a correlate for protection (31). Chiacchio et al. characterized the T cell response toward HBHA among human immunodeficiency virus-infected and -uninfected patients with LTBI and aTB and showed IFN-γ production by CD4 T cells to be generally lower in the human immunodeficiency virus-infected individuals (25). Dreesman et al. reported stimulation with HBHA in children and found that antigen induced CD4 T cells producing at least one cytokine to be significantly higher in both LTBI and aTB. Healthy children in that study showed a negligible cytokine response. In addition they showed that HBHA induced IL-17-procuding CD4 T cells only in young children below 3 years of age with LTBI but not in aTB patients and older children, suggesting that age may also influence the performance of certain antigens (34).

### The starvation regulon

The starvation regulon are a set of genes upregulated by *M. tuberculosis* in response to nutrient deprivation (52). Two examples that have been tested in a number of studies included in this review are Rv2659 and Rv2660. In a study by Govender et al. long-term stimulation of PBMCs with Rv2659 and Rv2660 resulted in an increase of cytokine production and proportions of IFN-γ/TNF-α/IL-2-producing polyfunctional CD4 T cells in LTBI compared to aTB. However, differences were more distinct upon stimulation with ESAT-6/CFP-10 and BCG. No differences were seen for the CD8 T cell population as proliferation and cytokine expression was generally low (36). Two studies in Greenland looking at remote and recent LTBI found that stimulation with Rv2659 and Rv2660 resulted in variable IFN-γ responses over the course of infection and were not associated with protection against disease progression. Furthermore, positive responses for those two antigens were also frequently observed in patients that tested negative in commercial interferon gamma-release assays, which questions the reliability of those findings (43, 44).

### *M. tuberculosis* reactivation-associated antigens and resuscitation promoting factors

Kassa et al. and Chegou et al. assessed immune responses to five Rpfs including Rv0867c, Rv1009, Rv1884c, Rv2389c, Rv2450c (26, 39). Stimulation with all five Rpfs induced significantly higher IFN-γ responses in household controls compared to aTB index cases. The two “reactivation-associated antigens” Rv1131 and Rv1471 were frequently recognized in household controls and showed significant differences resulting in increased INF-γ production in household contacts compared to aTB (26). Additional markers and cytokines were explored in a follow-up study and showed the diagnostic potential of Rv2389c-specific TGF-α concentrations being the most promising in discriminating aTB (27). Kassa et al. showed four Rpfs to be the most immunogenic and found high concentrations of additional cytokines (e.g., IL-6, IL-10, and TNF-α) in aTB patients (39). A study in Colombia showed that T cell responses toward Rv2389c were both mono- or bifunctional with increased frequencies of IFN-γ and/or TNF-α-producing CD4 and CD8 T cells in LTBI compared to aTB patients (21). Pathakumari et al. were able to demonstrate the discriminatory potential of Rv0753c specific IFN-γ and IFN-γ /TNF-α responses to identify LTBI and aTB patients (46).

### Other *M. tuberculosis* antigens

#### Region of difference encoded antigens

Dosanjh et al. investigated the role of several region of difference 1 encoded antigens in an ELISPOT assay in a study among children that were household contacts. Rv3873, Rv3878, and Rv3879c were included as additional antigens to improve sensitivity of the standard ELISPOT assay. Rv3873 and Rv3878 were found to be predictors of disease progression from LTBI to aTB (33).

Chen at al. tested a number of region of difference 2- and region of difference 11-encoded antigens for their potential of diagnosing LTBI in a BCG-vaccinated population (28). The immune response toward Rv1978, Rv1980c, Rv1981c, Rv3425, Rv3429, and Rv1984c was tested in an ELISPOT assay in LTBI and aTB patients as well as healthy controls. Rv1980c induced the highest frequency of IFN-γ producing T cells. However, none of the novel antigens tested did improve sensitivity compared to the commercial ELISPOT assay. Combining the read-out including Rv3425, Rv1981c and the region of difference1-encoded antigens (ESAT-6, CFP-10) however increased sensitivity for detecting aTB.

### Antigens of the Ag85 complex (Ag85A, Ag85B, Ag85C)

Antigens of the Ag85 complex (Ag85A, Ag85B, Ag85C) have been extensively studied and were included in a number of studies selected in this review. In the newer studies those antigens were mostly included as “classical” and “reference” antigens (29, 39, 41, 48), however three older studies, recruiting patients in South America reported more in-depth results (18, 19, 49). Schwander et al. reported stimulation with Ag85A and Ag85B resulted in significantly higher IFN-γ producing T cells in both PBMCs and broncho-alveolar cells of household contacts in a short-term assay compared to healthy controls (49). Antas et al. measured the immune response after stimulation with Ag85A and Ag85B in the course of anti-mycobacterial treatment in aTB patients and found IFN-γ concentrations to be higher in treated patients and community controls compared to untreated patients (19). Alvarez-Corales et al. reported that in addition to IFN-γ also IL-17 production was increased in exposed non-household contacts compared to aTB patients when stimulated with Ag85A and Ag85B (18).

### Other antigens

The secreted protein Rv1860 was found to induce higher frequencies of polyfunctional T cells dominated by a CD8 immune response in LTBI compared to aTB patients in a study in India (48).

Alvarez-Corrales explored the potential of several Pro-Pro-Glu family members in aTB patients and exposed healthcare workers (18). Rv3347 was a strong inducer of IFN-γ production in exposed individuals compared to aTB. On the other hand, IL-17 production was significantly higher in the exposed group compared to both aTB and healthy controls for the two antigens Rv0978c and Rv1917c (18).

Li et al. found that the inclusion of Rv3615c as addition in the commercial ELISPOT test improved sensitivity in detecting aTB patients. However, specificity was slightly lower compared to the commercial T-SPOT.TB test and most importantly the study lacks the inclusion of a LTBI patient group (40).

Pathakumari et al. investigated the role of the secreted proteins Rv0009 and Rv2204c among LTBI and aTB patients in a long-term assay. In both studies they looked at several cytokines and found that IFN-γ and IFN-γ /TNF-α responses to be the most accurate in identifying LTBI (45, 46).

The secreted antigens Rv0455c, Rv1511, and Rv1626 showed promise in a study measuring changes in immune response toward several antigens over treatment course. Bertholet et al. found that these secreted antigens showed changes in at least two different cytokines (IFN-γ, TNF-α, or IL-10) in aTB and associated this with treatment success (24).

## Discussion

Our review is, to our knowledge, the first to systematically review and summarize published evidence on the use of novel antigens for the diagnosis of TB. Although a large number of studies were screened for this review, 34 studies only were included in the final analysis. The main reason leading to the exclusion of many of the screened studies was the lack of detailed information in the particular studies relating to study population characteristics such as age, gender and immune status of the participants. In addition, clear disease classification for both LTBI and aTB was commonly missing as well as information whether patients have been started on treatment before the novel antigens were tested and what the prior duration of treatment was. Stringent inclusion criteria were therefore used in this review to enable comparison between studies and summarize evidence on novel antigens most promising to improve diagnosis before treatment initiation.

One of the most striking findings is that most of the tested novel antigens belong to the group of latency associated antigens, in particular from the DosR regulon. The DosR regulon is a specific region of the M. *tuberculosis* genome comprising approximately 50 genes that are activated during the dormant, non-replicative state (53). In general, the observed immune responses induced by these antigens are more pronounced in patients with LTBI compared to aTB, making these antigens attractive for distinction of infection vs. disease (54). Further to their potential for discrimination of LTBI and aTB these antigens may also be useful for monitoring treatment success in aTB patients.

The most promising candidate *M. tuberculosis* DosR regulon encoded antigens were Rv0081, Rv1733c, Rv1737c, Rv2029c, Rv2628, which showed high immunogenic potential across studies and geographical regions (20–22, 26, 27, 29, 35, 38, 39, 42).

Rv0081 is a transcriptional regulator (55) and presumably a key locus within the DosR regulon under hypoxic conditions (56). Several studies using long-term incubation in South Africa and Ethiopia showed the immunogenic potential of this antigen (26, 27, 39). However, Kassa et al. only included one group—being aTB patients—and both studies by Chegou et al. did not report tuberculin skin test or interferon gamma-release assays results for the LTBI patient groups. Hozumi et al. showed good immunogenic potential but addition of Rv0081 in a classic ELISPOT assay did not result in IFN-γ responses able to differentiate LTBI vs. aTB (38).

Rv1733c is presumed to be a transmembrane protein and found to be a highly potent T cell antigen using bioinformatic analysis by Zvi et al. (15) and Lew et al. (57). Indeed, the studies in our review show that this antigen elicits a higher immune response in LTBI patients compared to aTB and healthy controls. This is in agreement with serval other studies, which were excluded from the review, that the immune response to Rv1733c is potentially a good marker for LTBI patients (54, 58–60). However, only the study of Serra-Vidal et al. was able to show the discriminatory potential of Rv1733c both in short- and long-term stimulation assays (60).

Rv1737c is a possible nitrate transporter (61). Four studies in this review assessed its immunogenicity in long-term stimulation assays showing increased response in LTBI (21, 26, 27) but not aTB patients (39). An elevated immune response in LTBI patients was also described in further studies excluded from the review (62, 63) including in two studies investigating exposed individuals across several African sites (58, 64).

Rv2029c is thought to be a phosphofructokinase involved in glycolysis (65) and has been tested in mice as potential vaccine candidate antigen (66). It was one of the most widely used antigens among the studies in our review and the only antigen with concordant results across studies in short- and long-term assays. This is in agreement with several other studies not included in the review (54, 62–64).

Rv2628 was amongst the top ranking 45 antigens from a list of 189 antigens by Zvi et al. (15). However, its function is not yet clear (57). Many studies in this review employing a variety of different assays showed increased immune responses in LTBI compared to aTB patients (20, 22, 35, 42). Results from other studies not included in this review further highlight the importance of this antigen (54, 62, 63).

A further antigen from the DosR regulon is Rv2031c, also called α-crystallin or heat shock protein X, which has been described as crucial for growth of M. *tuberculosis* inside macrophages during latency (67, 68). Studies in mice and macrophage models have demonstrated its role during M. *tuberculosis* infection (67, 69, 70). The findings of the human studies included in our review are less clear with some studies reporting significantly higher cytokine production in exposed individuals and LTBI compared to aTB patients and controls.

Beyond the DosR regulon HBHA was a further latency associated antigen included in several studies for diagnosis of LTBI. HBHA is a protein that mediates dissemination of TB through its binding to epithelial cells at the site of primary infection, a process believed to be key for developing latency (71, 72). Concentrations of cytokines were significantly higher in LTBI compared to aTB patients in a number of studies (25, 30, 31, 34, 37, 41, 50, 51). Further to this a few studies including human immunodeficiency virus-infected individuals showed low or absent response to HBHA which may correlate with the risk for disease progression (25, 31). Interestingly in a study not included in this review HBHA-induced IFN-γ was detectable at the site of infection of TB disease in the absence of a response in the blood, suggesting that novel antigens might also be useful as stimulating antigens in samples from other body sites. As seen for several other antigens the HBHA-specific cytokine readout likely plays an important role as shown in a study by Molicotti et al. in which the ratio of IFN-γ and TNF-α was able to distinguish LTBI from aTB patients (73).

Further to the latency associated antigens a smaller number of studies evaluated Rpfs for *in-vitro* stimulation. Rpfs are a group of proteins involved in the reactivation of non-growing mycobacteria. The proteins are attached to the *M. tuberculosis* cell wall or secreted rendering them an optimal target for the immune system (74). The studies included in our review suggest that the immune response toward Rv0867c and Rv2389c are potent discriminators of disease stages. Reactivation is a key topic in TB research and therefore the number of studies investigating these antigens included in this review is surprisingly low. It is important to note that a few further studies investigating these antigens were not included as they did not fulfill the inclusion criteria. Results from the non-included studies showed similar results with Rv2389c (60, 63, 75, 76) and Rv0867c (63, 64, 75, 76) being identified as potent inducers of IFN-γ responses in LTBI compared to aTB patients and healthy controls.

Many studies report results using antigens from the Ag85 complex (Ag85A, Ag85B, Ag85C) with consistently elevated cytokine responses in LTBI compared to aTB. The Ag85 complex consists of three secreted proteins that are associated with virulence and crucial for survival of *M. tuberculosis* in macrophages (77). Due to its immune-dominant ability Ag85 has gained interest in vaccine research in recent years. There are currently vaccine candidates in clinical research mostly combining the existing BCG vaccine and additionally overexpressing a protein of the Ag85 complex (78). If successful as vaccines, the inclusion of one of these proteins in a novel diagnostic test could therefore interfere with the diagnosis as a result of cross-reactivity in a vaccinated population.

Apart from the inclusion of novel antigens, many studies extend the measurement of cytokines beyond IFN-γ to improve diagnostic assays (79, 80). Approximately half of the studies included in this review measured additional cytokines with IL-2, IL-10, IL-17, and TNF-α being among the most frequently used ones that improved diagnostic potential. A recent study by Coppola et al, not reviewed here, provided evidence that almost half of the new *M. tuberculosis* antigens tested in LTBI populations did trigger many other cytokines than IFN-γ, and often no IFN-γ itself (16).

One limitation of our review is that despite stringent inclusion criteria, there is inevitable variability between studies particularly for the patients included in the study groups. Whereas, the definitions for aTB patients were mostly consistent, there is considerable heterogeneity in the definition of LTBI patients. This often resulted from local routine practice of standard LTBI diagnosis. One further limitation is that some of the antigens have already been tested extensively and information on function and location are available. For others this information is still unknown, which renders interpretation of study results speculative in some instances.

## Conclusions

In our review compiling the latest research from novel *M. tuberculosis* antigens several studies clearly showed the discriminatory potential of LTBI from aTB of a number of antigens with the most promising being latency associated antigens. Findings for these antigens are consistent across several studies with immune responses detectable in short-term incubation periods. Moreover, the inclusion of additional cytokine read-outs complementing IFN-γ results, appears to increase discriminatory potential of LTBI from aTB. Despite these considerable advances in recent years especially children and immune-compromised patients are highly underrepresented in these studies and further research investigating novel antigens in these patient groups need to address this issue.

## Author contributions

NM designed the search strategy. NR reviewed and approved the search strategy. NM searched the literature, selected the studies, extracted, and analyzed the data. NR controlled the quality of the review process. NM drafted the initial manuscript. NR, MJ, and TO reviewed and edited the manuscript. All authors read and approved the final manuscript.

### Conflict of interest statement

The authors declare that the research was conducted in the absence of any commercial or financial relationships that could be construed as a potential conflict of interest.

## Abbreviations

CFP-10: 10 kDa culture filtrate protein
DosR: dormancy of survival regulon
ELISA: enzyme-linked immunosorbent assay
ELISPOT: enzyme-linked immuno-spot assay
EPTB: extrapulmonary tuberculosis
ESAT-6: 6 kDa early secretory antigenic target
FCM: flow cytometry
HBHA: Heparin-binding haemagglutinin
LTBI: latent tuberculosis infection
*M. tuberculosis*: *mycobacterium tuberculosis*
PBMC: peripheral blood mononuclear cell
QFT-GIT: quantiferon tuberculosis gold in tube
Rpf: resuscitation promoting factor
Rv: rough morphology virulent
TB: tuberculosis.
