# Supplementary material for: A Systematic Review on Novel Mycobacterium tuberculosis Antigens and Their Discriminatory Potential for the Diagnosis of Latent and Active Tuberculosis
Source: Front Immunol. 2018 Nov 9;9:2476. doi: 10.3389/fimmu.2018.02476 (PMC6237970; doi:10.3389/fimmu.2018.02476)
Supplement: Supplementary file 1 [file Table_1.docx]

**Supplementary Material**

**File 1: PRISMA checklist**

| **Section/topic** | **#** | **Checklist item** | | **Reported on page #** | |
| --- | --- | --- | --- | --- | --- |
| **TITLE** | | | |  | |
| Title | 1 | Identify the report as a systematic review, meta-analysis, or both. | | 1 | |
| **ABSTRACT** | | | |  | |
| Structured summary | 2 | Provide a structured summary including, as applicable: background; objectives; data sources; study eligibility criteria, participants, and interventions; study appraisal and synthesis methods; results; limitations; conclusions and implications of key findings; systematic review registration number. | | 1 | |
| **INTRODUCTION** | | | |  | |
| Rationale | 3 | Describe the rationale for the review in the context of what is already known. | | 2 | |
| Objectives | 4 | Provide an explicit statement of questions being addressed with reference to participants, interventions, comparisons, outcomes, and study design (PICOS). | | 2 | |
| **METHODS** | | | |  | |
| Protocol and registration | 5 | Indicate if a review protocol exists, if and where it can be accessed (e.g., Web address), and, if available, provide registration information including registration number. | | NA | |
| Eligibility criteria | 6 | Specify study characteristics (e.g., PICOS, length of follow-up) and report characteristics (e.g., years considered, language, publication status) used as criteria for eligibility, giving rationale. | | 2 | |
| Information sources | 7 | Describe all information sources (e.g., databases with dates of coverage, contact with study authors to identify additional studies) in the search and date last searched. | | 2, 3 | |
| Search | 8 | Present full electronic search strategy for at least one database, including any limits used, such that it could be repeated. | | Supplementary file 2 | |
| Study selection | 9 | State the process for selecting studies (i.e., screening, eligibility, included in systematic review, and, if applicable, included in the meta-analysis). | | 2, 3 | |
| Data collection process | 10 | Describe method of data extraction from reports (e.g., piloted forms, independently, in duplicate) and any processes for obtaining and confirming data from investigators. | | 5 | |
| Data items | 11 | List and define all variables for which data were sought (e.g., PICOS, funding sources) and any assumptions and simplifications made. | | 5 | |
| Risk of bias in individual studies | 12 | Describe methods used for assessing risk of bias of individual studies (including specification of whether this was done at the study or outcome level), and how this information is to be used in any data synthesis. | | NA | |
| Summary measures | 13 | State the principal summary measures (e.g., risk ratio, difference in means). | | NA | |
| Synthesis of results | 14 | Describe the methods of handling data and combining results of studies, if done, including measures of consistency (e.g., I^2^) for each meta-analysis. | | NA | |
| Section/topic | # | Checklist item | | Reported on page # | |
| Risk of bias across studies | 15 | Specify any assessment of risk of bias that may affect the cumulative evidence (e.g., publication bias, selective reporting within studies). | | NA | |
| Additional analyses | 16 | Describe methods of additional analyses (e.g., sensitivity or subgroup analyses, meta-regression), if done, indicating which were pre-specified. | | NA | |
| **RESULTS** | | | | |  |
| Study selection | 17 | | Give numbers of studies screened, assessed for eligibility, and included in the review, with reasons for exclusions at each stage, ideally with a flow diagram. | | 3, supplementary file 4 |
| Study characteristics | 18 | | For each study, present characteristics for which data were extracted (e.g., study size, PICOS, follow-up period) and provide the citations. | | 4-6 |
| Risk of bias within studies | 19 | | Present data on risk of bias of each study and, if available, any outcome level assessment (see item 12). | | NA |
| Results of individual studies | 20 | | For all outcomes considered (benefits or harms), present, for each study: (a) simple summary data for each intervention group (b) effect estimates and confidence intervals, ideally with a forest plot. | | NA |
| Synthesis of results | 21 | | Present results of each meta-analysis done, including confidence intervals and measures of consistency. | | NA |
| Risk of bias across studies | 22 | | Present results of any assessment of risk of bias across studies (see Item 15). | | NA |
| Additional analysis | 23 | | Give results of additional analyses, if done (e.g., sensitivity or subgroup analyses, meta-regression [see Item 16]). | | 7 |
| **DISCUSSION** | | | | |  |
| Summary of evidence | 24 | | Summarize the main findings including the strength of evidence for each main outcome; consider their relevance to key groups (e.g., healthcare providers, users, and policy makers). | | 18, 19 |
| Limitations | 25 | | Discuss limitations at study and outcome level (e.g., risk of bias), and at review-level (e.g., incomplete retrieval of identified research, reporting bias). | | 19 |
| Conclusions | 26 | | Provide a general interpretation of the results in the context of other evidence, and implications for future research. | | 20 |
| **FUNDING** | | | | |  |
| Funding | 27 | | Describe sources of funding for the systematic review and other support (e.g., supply of data); role of funders for the systematic review. | | 20 |

*From:*  Moher D, Liberati A, Tetzlaff J, Altman DG, The PRISMA Group (2009). Preferred Reporting Items for Systematic Reviews and Meta-Analyses: The PRISMA Statement. PLoS Med 6(7): e1000097. doi:10.1371/journal.pmed1000097

**File 2: Search strings for the three different databases**

**PubMed (Mesh terms)**

*#A Search string for Tuberculosis:*

"Tuberculosis/blood"[Mesh] OR "Tuberculosis/diagnosis"[Mesh] OR "Tuberculosis/epidemiology"[Mesh] OR "Tuberculosis/immunology"[Mesh] OR “Mycobacterium tuberculosis/immunology”[Mesh]

*#B Search string for antigens:*

"Antigens, Bacterial/immunology"[Mesh] OR "Bacterial Proteins/immunology"[Mesh]

*#C Search string for assay read out:*

"Interferon-gamma/blood"[Mesh] OR "Interferon-gamma/immunology"[Mesh] OR "Interferon-gamma/secretion"[Mesh] OR "Interferon-gamma/biosynthesis"[Mesh] OR "Cytokines/secretion"[Mesh] OR "Cytokines/blood"[Mesh] OR "enzyme-linked immunosorbent assay"[MeSH]

*#D Search string for Humans:*

"Humans"[Mesh]

Search: #A AND #B AND #C AND #D

**EMBASE**

*#A Search string for Tuberculosis:*

Tuberculosis OR lung tuberculosis

*#B Search string for antigens:*

antigen OR bacterial antigen OR antigen recognition OR Mycobacterium antigen OR bacterial protein OR antigen expression OR immuno stimulation OR enzyme linked immunosorbent assay

*#C Search string for assay read out:*

Gamma interferon OR cytokine production OR cytokine response OR immune response

*#D Search string for Humans:*

Human

Search: #A AND #B AND #C AND #D

Additional filters:

Publication types:

- articles

Diseases:

- tuberculosis
- lung tuberculosis
- latent tuberculosis
- tuberculous pleurisy
- tuberculous meningitis
- extrapulmonary tuberculosis
- mycobacteriosis

Floating subheadings:

- diagnosis
- prevention
- epidemiology
- clinical trial

**Web of Science (core collection)**

*#A Search string for Tuberculosis:*

TS=(tubercu* OR mycobac* tubercu*)

*#B Search string for antigens:*

TS=(antigen* stim* OR bact* antigen*)

*#C Search string for assay read out:*

TS=(interferon-gamma* OR IFN-gamma OR cytok* OR enzyme-linked immunosorbent assay OR ELISA)

*#D Search string for Humans:*

TS=(human*)

Search: #A AND #B AND #C AND #D

Additional filters:

Publication types:

- articles

# File 3: Inclusion and exclusion criteria

Systematic review inclusion and exclusion criteria

1. Study design
   1.1 Patients untreated or treated for a maximum of 4 weeks (unless separate analysis)
   1.2 Well defined study population (including age, gender, human immunodeficiency virus status)
   1.3 Well defined diagnosis of TB infection and disease

1.4 TB infection and/or TB disease group

1. Antigens
   2.1 Inclusion of at least one additional antigen not used in commercial tests (ESAT-6, CFP-10, TB7.7, purified protein derivative)

2.1 studies including use of genomic regions rather than distinct antigens excluded

1. Outcome
   3.1 Cellular immunity only
   3.2 Humoral immunity excluded

3.3 If human immunodeficiency virus patients included, separate analysis must be given

1. Others

4.1 Papers written in anything other than English, German or French excluded

4.2 reviews excluded

4.3 only studies involving humans included

**File 4: Reasons for exclusion of screened articles**

| **Reason** | **Title screen** | **Abstract screen** | **Full-text screen** | **Data extraction** | **Total** |
| --- | --- | --- | --- | --- | --- |
| **Duplicates** | 134 | - | - | - | 134 |
| **Non-human studies** | 92 | 12 | 1 | - | 105 |
| **In-vitro studies** | 463 | 47 | 11 | - | 521 |
| **No novel antigens** | 196 | 71 | - | - | 267 |
| **Non M. tuberculosis antigens** | 117 | 2 | 1 | - | 120 |
| **TB antibodies** | 84 | 50 | 5 | - | 139 |
| **Vaccine specific** | 92 | 5 | 9 | - | 106 |
| **Review, Letter** | 40 | 1 | 4 | - | 45 |
| **Language** | - | - | 1 | - | 1 |
| **Full text not available** | 7 | - | 1 | - | 8 |
| **Study population issues** | - | - | 28 | 31 | 59 |
| **Total** | 1225 | 188 | 61 | 31 | **1505** |
